# A Longitudinal Multimodal Dataset of Type 1 Diabetes

**DOI:** 10.1038/s41597-025-05695-1

**Published:** 2025-08-07

**Authors:** Ashwaq Alsuhaymi, Ahmad Bilal, Daniel Gasca García, Rujiravee Kongdee, Nicole Lubasinski, Hood Thabit, Paul W. Nutter, Simon Harper

**Affiliations:** 1 Department of Computer Science, University of Manchester, Manchester, M13 9PL UK; 2 Division of Diabetes, Endocrinology and Gastroenterology, Faculty of Biology, Medicine and Health, University of Manchester, Manchester, UK

**Keywords:** Type 1 diabetes, Diabetes complications

## Abstract

People living with Type 1 Diabetes (PwT1D) must continuously monitor blood glucose levels and make critical clinical and safety-related decisions multiple times a day to maintain glycaemic control within recommended ranges. While significant efforts have been made to develop algorithms that assist PwT1D in managing blood glucose more effectively, access to automated insulin delivery (AID) systems remains highly variable across the world. Moreover, there is a lack of publicly available, comprehensive datasets necessary for developing algorithms to support scenarios where AID systems revert to manual mode. This study addresses this gap by providing a detailed, multimodal dataset encompassing five key aspects: blood glucose levels; basal and bolus insulin dosages; nutritional intake (carbohydrates, protein, fat, and fibre content); physical activity (step count, active calories, distance covered, MET, and intensity level); and sleep patterns. The dataset includes longitudinal (3-month) real-world data collected from 17 PwT1D participants. By making this resource available, the study aims to advance algorithm development and improve diabetes management, particularly in settings where AID technology is less accessible.

## Background & Summary

Type 1 Diabetes (T1D) is a chronic autoimmune disorder that destroys pancreatic beta cells, resulting in a loss of insulin production and the body’s inability to self-regulate blood glucose levels (BGL)^1^. In the United Kingdom (UK), diabetes affects roughly 8% of the population, with approximately 10% of these cases classified as T1D, according to the Breakthrough T1D^2^.

Managing diabetes places a substantial financial burden on healthcare resources; nearly 10% of the annual budget of the National Health Service (NHS) in England and Wales is allocated to diabetes care in general^3^. However, when it comes to T1D, access to advanced technologies such as closed-loop insulin delivery systems is becoming more common in high-income countries, while remaining limited in low- and middle-income countries (LMICs) and among individuals without sufficient health insurance coverage^4^.

Chronic complications arising from poor glycaemic control significantly heighten health risks and mortality in PwT1D compared to non-diabetic individuals, making strict glycaemic control essential for mitigating this risk^5^. Consequently, management of T1D requires PwT1D to make multiple daily decisions on monitoring their BGL, administering correct insulin dosages and managing hypo- or hyper-glycaemia when they occur. Several types of technologies, such as wearable glucose sensors and insulin pump devices, have been developed to help PwT1D improve their glycaemic control while minimally impacting the patient’s quality of life^6^. However, despite this, less than 40% of PwT1D achieve the recommended level of glycaemic control required to reduce the risk of complications^7^.

Research indicates that reliable blood glucose predictions could significantly improve the quality of life of PwT1D^8^. Consequently, intelligent diabetes management systems require BGL prediction algorithms that accurately mimic daily glycaemic variability while responding to the spontaneity of everyday life^6^. These predictions must respond to the various factors that affect BGL^9^, including insulin administration, food intake (carbohydrate), physical activity, and sleep patterns^10^.

The current state-of-the-art technology for T1D management is automatic insulin delivery (AID), also known as closed-loop systems^11^. These systems automate insulin dosing based on continuous glucose monitoring; however, they can temporarily switch to manual mode—standard insulin pump operation—in cases of connectivity issues or specific manufacturer-defined conditions^12^. This study provides a comprehensive dataset capturing real-world data from PwT1D, which could also facilitate the development of algorithms to support clinicians in LMICs, where AID remains less prevalent, expensive, or unavailable.

Publicly available datasets with similar variables, such as HUPA-UCM^13^, Tidepool^14^, diaTribe^15^, and OhioT1DM^16^, provide valuable insights into diabetes management. However, the dataset presented here offers unique advantages as it offers a more comprehensive analysis of long-term glycaemic trends and lifestyle factors. Unlike the 14-day HUPA-UCM dataset, which relies on the Fitbit Ionic and lacks Metabolic Equivalent of Tasks (METs) and motion intensity tracking, our dataset spans three months and leverages the Garmin Forerunner 45 to capture detailed activity data, including step count, calories burned, distance, METs, motion intensity, and categorized activity types. This richer set of physiological parameters allows for more detailed insights into activity-related blood glucose variability. Additionally, while HUPA-UCM is limited to FreeStyle Libre 2 data, our dataset integrates data from multiple continuous glucose monitor (CGM) platforms (LibreView, Dexcom, and Medtronic). Similarly, the Tidepool dataset aggregates real-world diabetes data from CGMs, insulin pumps, and manual log entries, providing patient-centered insights. In contrast, the diaTribe dataset primarily focuses on educational and research-based data, often derived from surveys and expert analyses rather than structured numerical datasets. The OhioT1DM dataset, specifically designed for T1D research, includes CGM data, insulin administration, carbohydrate intake, and other physiological factors, making it an essential resource for machine learning applications in glucose prediction and personalised treatment strategies. Our dataset expands on the commonly used nutritional information for meals consumed by including carbohydrate, fat, protein, and fibre content, along with a simple descriptor of each meal. This allows for a more nuanced understanding of the impact of macronutrient composition on blood glucose dynamics. By combining the strengths of existing datasets and addressing their limitations, our dataset contributes to advancing diabetes research, improving predictive modelling, and enhancing patient care strategies.

Compared to prominent datasets such as OhioT1DM and HUPA-UCM, our dataset offers distinctive advantages in demographic diversity, temporal resolution, and multimodal richness. The OhioT1DM dataset comprises 12 adult participants (8 male, 4 female) but lacks BMI data and exhibits limited age variability. While it includes pump-based insulin delivery and structured meals, the coverage of continuous glucose monitoring (CGM) and physical activity data is inconsistent between its two cohorts, and detailed nutritional information is sparse. Notably, physical activity data in OhioT1DM is not available for the full day. The HUPA-UCM dataset, on the other hand, involves 10 individuals aged approximately 20 to 50 years, monitored over 14 days using Freestyle Libre and Fitbit devices. Although it emphasises physical activity, it lacks comprehensive insulin records, precise meal composition, and objective intensity metrics such as metabolic equivalents (METs) or gradient. Activity levels are based on Fitbit classifications, and both meal and insulin data are self-reported, without validation of temporal alignment. In contrast, our dataset comprises 17 participants, balanced by gender (10 female, 7 male), spanning a broader age range (23–70 years) and includes documented BMI values (20.3–36.5 kg/m^2^). It offers 12 weeks of high-resolution, objectively captured data across six modalities, including Garmin-derived step counts, intensity levels, and sleep staging. This unique combination of demographic breadth, longitudinal depth, and sensor-derived multimodal data provides an unprecedented opportunity for personalised modelling of glycaemic dynamics under free-living, real-world conditions.

This dataset includes participants using both multiple daily injections (MDI) and insulin pumps operating in open-loop mode. These insulin delivery methods differ in dosing flexibility and associated glycaemic outcomes, with pump users often exhibiting distinct Time in Range (TIR) profiles compared to MDI users—primarily due to the increased adaptability of pump therapy rather than automation, as shown in previous studies^17,18^. The inclusion of multiple delivery modalities supports the evaluation of algorithm performance across diverse real-world treatment scenarios and enables stratified analyses by reported delivery method, particularly when combined with continuous glucose monitoring (CGM) metrics. This is especially relevant in contexts where access to closed-loop systems remains limited. Variability in insulin delivery methods and TIR should be carefully considered when developing and validating predictive models using these data.

The longitudinal nature of the dataset presented here—spanning a 12-week (90-day) period—captures sustained patterns in blood glucose levels (BGLs) alongside relevant lifestyle factors. This extended duration aligns with the time frame over which HbA1c, a key biomarker for longer-term glucose control, is typically measured (8–12 weeks)^19^. Because HbA1c reflects average glucose over several weeks, rather than short-term fluctuations, the dataset offers a valuable resource for exploring how real-world behaviours and glucose trends may relate to HbA1c outcomes. Studies have shown that sampling bias in shorter periods, such as 10 days, can be as high as 47%, decreasing substantially to 26.4% after 30 days^20^, further highlighting the importance of longer-term data for accurate assessment and prediction. A 12-week data window meets most regulatory requirements in treatment evaluation and is a standard duration used in phase II clinical trials for diabetes drugs, allowing researchers to draw meaningful comparisons of interventions and their sustained effects on blood glucose management^21^.

Further distinctions of the dataset includes more precise insulin tracking, with separate basal and bolus data being provided, compared to HUPA-UCM that resamples insulin data at five-minute intervals, reducing accuracy. Our dataset also provides standardized nutritional data via Nutritics^22^, offering detailed macronutrient breakdowns (Fig. 8(a–d)), whereas HUPA- UCM records carbohydrates in “servings.” Additionally, HUPA-UCM sleep data is organized by night, with separate files that detail the start times and durations spent in each sleep stage based on Fitbit’s general sleep scores. In contrast, our sleep data provides a similarly structured stage breakdown but includes additional granular details about the transitions and specific dynamics of each sleep stage, offering a deeper insight into sleep patterns. These advantages make our dataset better suited for real-world diabetes management and artificial intelligence (AI)-driven glucose prediction, integrating a comprehensive range of parameters over a clinically relevant period. In contrast, HUPA-UCM, while useful for short-term glucose variation analysis, lacks the depth and granularity needed for extensive diabetes research.

## Methods

Following a longitudinal observational design, with data collection spanned from 1 October 2023, to 3 September 2024. Participants were recruited online through the social media pages of the Interaction Analysis and Modeling Lab (IAM Lab) group, T1D-specific social media groups, email outreach, broad-reaching tweets, social media posts, and physical advertisements placed in several buildings on the University of Manchester campus and in nearby locations such as sports centres. Potential participants were given at least 24 hours to consider their involvement to ensure a non-coercive recruitment process. PwT1D over the age of 18, who had been living with T1D for more than two years and who used CGM, were invited to participate. Applicants were excluded from the data collection if they had additional conditions that impacted their nutritional intake or if they used medications that affected their sleep and/or physical performance. Detailed inclusion and exclusion criteria can be found in Table 1. After an initial screening, eligible participants were contacted for a face-to-face/online interview conducted by researchers from the study team. During this interview, participants received instructions on recording their nutritional intake and how to wear the smartwatch (Garmin Forerunner 45) to ensure comprehensive data collection, including sleep tracking. Informed consent was also obtained at this stage for accessing blood glucose sensor and insulin device platforms. The participants were then issued a smartwatch for the study and at the end of the data collection period, additional consent was obtained to access the watch data.

**Table 1:** Inclusion and Exclusion Criteria for Study Participants.

Inclusion Criteria	Exclusion Criteria
PwT1D over the age of 18	Presence of sleep apnoea or co-morbidities (e.g., cancer, kidney disease, Crohn’s disease) that impose additional nutritional re- strictions
Diagnosed with T1D for over 2 years	Pregnancy, due to its impact on glycaemic control
Regular use of prescribed glucose sensors	Shift worker, as it significantly alters sleep patterns
	Use of medications affecting sleep and/or physical performance (e.g., sleep tablets, melatonin, performance-enhancing drugs)
	Reliance on Self-Monitoring of Blood Glucose (SMBG) via a glucometer instead of a continuous glucose sensor

### Ethical approval

This study was reviewed and approved by the University of Manchester Research Ethics Committee before data collection began (Ref: 2023-15687-29584). Data collection adhered to all legal requirements and followed the principles of the Declaration of Helsinki, Good Clinical Practice (GCP), and the UK Policy Framework for Health and Social Care Research 2017. All participants provided informed consent for their data to be published.

### Data collection

At the beginning of the study, all participants were instructed not to change their lifestyle or make any adjustments to their food intake, physical activity, or sleep patterns. This ensured that the data collected reflected their usual behaviours without external influences.The 12-week period was selected to align with the timeframe reflected by HbA1c measurements, and data collection commenced shortly after participants had a clinically measured HbA1c value. This alignment ensured that consistent lifestyle patterns could be observed throughout a period directly relevant to long-term glucose control.

#### Blood glucose data collection

Participants were already using CGM sensors as part of their routine clinical care. and were linked to LibreView^23^, Dexcom Clarity^24^ and Medtronic Carelink platforms^25^. Participants provided informed consent for their CGM data to be downloaded and analysed.

#### Insulin data collection

Participants on insulin pump (Tandem t: slim X2^26^, MiniMed 780 G^25^, and Omnipod 5^12^) as part of their routine clinical care had insulin delivery data downloaded from their respective device platforms and exported in CSV format. These files contained detailed information, including timestamps, insulin types (e.g., bolus or basal), and dosage amounts. Those on multiple daily insulin pen injections (MDI) electronically recorded their insulin data on platforms, such as the FreeStyle LibreLink app^27^ and Dexcom G6^28^, which were then exported in CSV format.

#### Nutrition data collection

Participants were able to choose from two methods to record food intake. The first option was to use the mobile application, MyFitnessPal^29^, which allows for commonly consumed foods to be logged and tagged with associated recipes or ‘my foods’ options. Alternatively, participants could opt for a manual food diary, which required recording the time of the meal, meal type, foods consumed, estimated carbohydrate content, and insulin administered, alongside the corresponding food tag for each meal. Given the variability in how food diaries were maintained, all entries were standardised using Nutritics^22^ to ensure consistency in nutritional analysis across datasets. Participants were instructed to comprehensively document their food intake along with a food tag, a single descriptive word for each meal, for the first two weeks of the data collection period. Thereafter, only the food tag and detailed information on any newly introduced foods were required for each meal and snack. Each tag was unique to a specific meal and could be any identifier, provided it consistently referred to the same food item. For instance, different types of breakfast cereal would require distinct tags, such as ‘cornflakes’ or ‘muesli.’ The tags could be hyphenated (e.g., ‘fruit-yogurt’) and did not need to fully describe the food consumed, being as simple as ‘breakfast1’ or ‘breakfast2’ providing they were specific to a particular meal or snack.

#### Activity data collection

Participant activity data was collected using the Garmin Forerunner 45 smartwatch. A custom API was developed for this project (Bilal, A., https://iam-research.manchester.ac.uk/flaskapp/) to seamlessly integrate with the Garmin Connect Developer Program, enabling real-time, historical, and batch data retrieval. This API captured detailed raw activity data, including HEALTH - Epochs, which provides a structured time-series dataset. Developed using a Python framework, the API was deployed on The University of Manchester server. Participants received a secure access link and provided consent via the Garmin API Developer Platform to share their data for collection and analysis.

#### Sleep data collection

Each participant was instructed to wear the Garmin Forerunner 45 continuously throughout the study, including during sleep, removing it only for charging. Sleep data were retrieved through the Garmin Connect app using a dual approach to ensure comprehensive data collection. Participants first downloaded and shared all sleep-related data directly from their Garmin Connect app accounts. Additionally, sleep data were accessed via the Garmin Connect API, with participants consenting to share their smartwatch data through their Garmin Connect credentials. This approach ensured the collection of detailed sleep metrics, including sleep stages, while maintaining data security and participant privacy.

## Data Records

The dataset is available in a Zenodo repository^30^ ‘T1D-UOM – A Longitudinal Multimodal Dataset of Type 1 Diabetes’ at; 10.5281/zenodo.15806142.

### Participant information

Twenty-one participants were initially recruited, however, four withdrew due to personal reasons. Data from the remaining 17 participants were available for the final analysis. Table 2 outlines the information regarding the participants’ demographics, start and end dates of data collection, and devices used.

**Table 2:** Participant demographics.

(a)							
Participant ID	Gender	Age (years)	Start Date	End Date	Sensor	Insulin device	TIR (%)
UoM2301	Female	25	6-Oct-2023	29-Dec-2023	CGM	Tandem t:slim X2	78.81
UoM2302	Female	29	1-Oct-2023	24-Dec-2023	Flash	MDI	87.81
UoM2303	Female	29	17-Oct-2023	9-Jan-2024	CGM	N/A	92.92
UoM2304	Female	29	17-Oct-2023	5-Feb-2024	CGM	MiniMed 780 G	71.17
UoM2305	Female	25	17-Oct-2023	9-Jan-2024	Flash	MDI	48.29
UoM2306	Female	50	18-Oct-2023	10-Jan-2024	Flash	MDI	82.83
UoM2307	Female	61	13-Oct-2023	5-Jan-2024	CGM	Tandem t:slim X2	67.80
UoM2308	Male	59	4-Dec-2023	26-Feb-2024	CGM	MiniMed 780 G	84.41
UoM2309	Female	59	5-Feb-2024	1-May-2024	CGM	MiniMed 780 G	54.29
UoM2310	Male	70	21-Jun-2023	12-Apr-2024	CGM	MiniMed 780G	89.26
UoM2313	Male	39	10-Nov-2023	2-Feb-2024	Flash	MDI	52.97
UoM2314	Male	61	6-Nov-2023	29-Jan-2024	Flash	MDI	64.77
UoM2320	Female	46	1-Dec-2023	23-Feb-2024	CGM	Omnipod 5	93.88
UoM2401	Male	46	19-Jan-2024	1-May-2024	Flash	MDI	70.16
UoM2403	Male	23	12-Mar-2024	25-Jun-2024	Flash	MDI	62.15
UoM2404	Female	37	11-Mar-2024	10-Jun-2024	Flash	MDI	71.37
UoM2405	Male	52	3-Jun-2024	3-Sep-2024	Flash	MDI	64.40
**(b)**							
**Participant ID**	**Glucose**	**Activity**	**Basal**	**Bolus**	**Nutrition**	**Sleep Time**	**Sleep**
UoM2301	x	x	x	x	x	x	x
UoM2302	x	x	x	x	x	x	x
UoM2303	x	x				x	x
UoM2304	x	x	x	x	x	x	x
UoM2305	x	x	x	x	x		x
UoM2306	x	x	x	x	x	x	x
UoM2307	x	x	x	x	x	x	x
UoM2308	x	x	x	x	x	x	x
UoM2309	x	x	x	x	x	x	x
UoM2310	x	x	x	x		x	x
UoM2313	x	x	x	x	x	x	x
UoM2314	x	x	x	x	x	x	
UoM2320	x	x		x	x	x	x
UoM2401	x	x	x	x	x	x	x
UoM2403	x	x	x	x	x	x	
UoM2404	x	x		x	x	x	x
UoM2405	x	x	x	x	x	x	x

(**a**) Demographics, sensor and insulin delivery method, and Time in Range (TIR). (**b**) Data availability across domains: glucose, activity, insulin (basal and bolus), nutrition, and sleep.

Certain data were unavailable from some participants due to technical issues or failure to submit the required information during the study period. Data from one participant (UoM2303) was unable to be collected as the participant had an unplanned trip abroad during the study period.

**Fig. 1:**
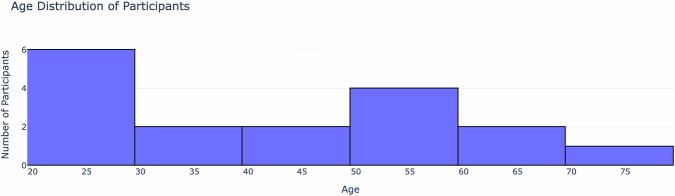
Age distribution of study participants. The figure illustrates the range and frequency of ages in the dataset, providing insight into the demographic composition of the cohort. Most participants were in the 20–30 age bracket, with only one participant aged 70 years.

Figure 1 illustrates the age distribution of participants. A balanced age range enhances the dataset’s applicability for research on T1D management, including personalized glucose prediction models, HbA1c trends, and the impact of lifestyle factors on long-term glycaemic control. By including both younger and older participants, researchers can develop AI-driven models that generalize across different age groups, accounting for variations in insulin sensitivity, metabolic rates, and physical activity patterns.

**Fig. 2:**
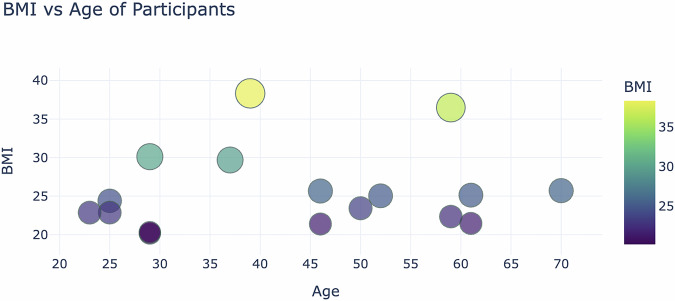
BMI vs. Age distribution of study participants. The figure displays the relationship between body mass index (BMI) and age, illustrating variations in body composition across different age groups. The youngest participant (23 years) has a BMI of 20.3 kg/m^2^, while the oldest participant (70 years) has a BMI of 25.7 kg/m^2^. The highest BMI in the dataset is observed in participant UoM2309 (36.5 kg/m^2^), whereas the lowest BMI is recorded for participant UoM2303 (20.3 kg/m^2^).

To explore the metabolic diversity within the dataset, Fig. 2 provides valuable context. This bubble graph illustrates the relationship between age and BMI, highlighting variation in body composition across participants. Such differences may influence insulin resistance, which is a key factor in personalised T1D management. Insights from this data can inform strategies for optimising insulin dosing, dietary recommendations, and physical activity guidelines tailored to different age groups.

The descriptive statistics of BGLs and activity information for all participants are provided in Tables 3, 4, respectively.

**Table 3:** Descriptive statistics of glucose levels for all participants, including mean glucose values, standard deviations, number of recorded glucose readings, and calculated days.

Participant ID	Mean Glucose (mmol/L)	Std Dev (mmol/L)	Total Readings	Observation Days
UoM2301	8.23	2.61	45,768	110
UoM2302	7.49	2.07	13,656	170
UoM2303	7.10	1.82	14,188	50
UoM2304	8.73	3.25	39,512	117
UoM2305	10.19	4.02	7,190	64
UoM2306	7.07	2.46	11,710	103
UoM2307	9.19	3.53	8,385	30
UoM2308	6.95	2.51	28,694	107
UoM2309	9.85	3.96	20,665	86
UoM2310	7.19	2.05	50,108	178
UoM2313	9.76	4.51	30,832	100
UoM2314	9.11	3.36	12,783	92
UoM2320	7.09	1.62	23,965	84
UoM2401	7.70	3.22	15,047	92
UoM2403	8.92	3.43	12,860	117
UoM2404	8.38	3.00	8,236	79
UoM2405	8.68	3.10	12,547	99

**Table 4:** Descriptive statistics of activity levels for all participants, including mean values, standard deviations, number of recorded activity readings, and the duration of observation in days.

Partcipant ID	active_Kcal_mean	active_Kcal_std	step_count_mean	step_count_std	distance_m_mean	distance_m_std	duration_s_mean	duration_s_std	active_time_s_mean	active_time_s_std	total_days
UoM2301	2.67	7.40	58.92	209.80	43.75	162.63	900.00	0.00	627.99	351.57	692
UoM2302	2.51	6.93	57.53	161.22	45.43	126.02	921.15	889.80	584.54	781.61	693
UoM2303	2.38	6.71	73.54	226.52	54.03	170.36	904.53	370.40	621.38	502.19	335
UoM2304	2.92	6.26	39.66	139.38	28.92	101.02	902.34	145.34	545.39	383.00	691
UoM2305	10.79	16.83	29.08	117.16	66.05	139.33	902.45	243.93	631.26	378.72	340
UoM2306	2.88	8.22	73.81	226.68	56.13	177.36	933.12	832.70	605.63	675.63	661
UoM2307	3.24	8.41	95.63	274.18	74.73	228.61	900.64	33.83	591.22	332.44	661
UoM2308	3.28	13.40	92.35	307.46	84.46	315.80	915.84	320.80	570.76	412.36	661
UoM2309	1.42	4.35	40.56	146.06	30.88	114.06	900.00	0.00	626.61	341.12	337
UoM2310	4.34	17.55	62.18	225.26	54.29	206.41	901.26	93.15	609.72	355.44	691
UoM2313	6.14	14.05	25.27	94.28	21.13	78.48	900.17	12.30	597.13	368.10	691
UoM2314	3.53	15.94	72.66	235.07	67.69	253.11	900.00	0.00	593.57	326.10	690
UoM2320	3.54	11.26	67.90	238.19	54.98	221.22	901.02	80.34	586.48	342.20	691
UoM2401	4.02	12.58	54.88	184.08	47.82	175.81	916.72	824.21	614.36	682.29	337
UoM2403	5.13	10.75	49.88	182.57	40.47	146.56	901.32	106.78	537.11	383.48	338
UoM2404	2.43	7.03	72.47	244.00	52.47	177.10	902.84	130.15	619.64	363.87	338

This includes mean values, standard deviations, number of recorded readings, and duration of observation in days.

### Dataset structure

The complete dataset is outlined in Table 5, which provides information of each subfolder in the UoMT1D Dataset folder. All files are in the comma-separated value (CSV) format, using a comma as the delimiter with UTF-8 encoding.

**Table 5:** Dataset structure.

Folder name	Number of files	Number of records	Folder size
Glucose Data	17 files	356,146	7.9 MB
Insulin Data	Basal Insulin: 14 files	20,407	608 KB
	Bolus Insulin: 16 files	5,660	188 KB
Nutrition Data	15 files	4,351	295 KB
Activity Data	17 files	228,681	17.8 MB
Sleep Data	Sleep time: 15 files	323,340	13.9 MB
	Sleep: 15 files	1,495	

### Blood glucose data

Table 6 shows the glucose data files overview. The *UoMGlucoseID.csv* is the file that includes two fields describing blood glucose data. The *bg_ts* field records the exact time of each observation in the format *MM/DD/YYYY HH:MM*, providing high-resolution temporal data crucial for monitoring blood glucose trends over time. The *value* field specifies the blood glucose reading as a floating-point value, measured in *mmol/L*, offering precise quantification of glucose levels. Table 7 provides example BGL data.

**Table 6:** UoM Blood Glucose Data Description.

Column	Type	Description	Units	Possible Values
bg_ts	Datetime	Datetime of observation	MM/DD/YYYY HH:MM	N/A
value	Float	Blood glucose reading	mmol/L	N/A

**Table 7:** Blood glucose data example of UoM2301.

bg_ts	value
01/10/2023 00:04	7.5

### Insulin data

The *UoMBasalID.csv* file includes three key fields describing basal insulin data, as shown in Table 8. The *basal_ts* field records the timestamp of each observation in the format *MM/DD/YYYY HH:MM*, capturing both date and time to enable precise temporal analysis. The *basal_dose* field specifies the basal insulin rate as a floating-point value, with units represented as either *U* (units) for participants using long-acting insulin or *U/h* (units per hour) for those using rapid-acting insulin, providing essential information on dosage for therapy monitoring. The *insulin_kind* field identifies the type of insulin administered, with possible values *R* (rapid-acting insulin) and *L* (long-acting insulin), facilitating differentiation between formulations used in treatment. Table 9 provides example basal insulin data. For an overview of which participants use each insulin type, see Table 21.

**Table 8:** UoM Basal Data Description.

Column	Type	Description	Units	Possible Values
basal_ts	Datetime	Datetime of observation	MM/DD/YYYY HH:MM	N/A
basal_dose	Float	Basal rate	U or U/h	N/A
insulin_kind	String	Kind of insulin	N/A	R/L

**Table 9:** Basal Insulin Data Example of UoM2301.

basal_ts	basal_dose	insulin_kind
10/11/2023 00:00	1.725	R

*UoMBolusID.csv* files includes two fields describing bolus insulin data, as shown in Table 10. The *bolus_ts* field captures the timestamp of each observation in the format *MM/DD/YYYY HH:MM*, enabling precise tracking of the timing of bolus insulin administration. The *bolus_dose* field specifies the bolus insulin dose as a floating-point value, with units recorded as *U* (units), providing detailed information on the administered dosage. Table 11 provides example bolus insulin data.

**Table 10:** UoM Bolus Data Description.

Column	Type	Description	Units	Possible Values
bolus_ts	Datetime	Datetime of observation	MM/DD/YYYY HH:MM	N/A
bolus_dose	Float	Bolus dose	U	N/A

**Table 11:** Bolus Insulin Data Example of UoM2301.

bolus_ts	bolus_dose
10/11/2023 12:43	2.36

### Nutrition data

The *UoMNutritionID.csv* file provides detailed information about nutritional data through six key fields, as shown in Table 12. The *meal_ts* field records the datetime of the observation in the format *MM/DD/YYYY HH:MM*, enabling precise tracking of meal timing. The *meal_type* field specifies the type of meal, with possible values including *Breakfast*, *Lunch*, *Dinner*, and *Snack*, allowing for categorisation of dietary intake. The *meal_tag* field briefly describes the food eaten, offering additional context about the meal’s composition. The *carbs_g* field quantifies the amount of carbohydrates consumed in grams, while the *prot_g* field records the amount of protein consumed in *grams*. Similarly, the *fat_g* and *fibre_g* fields measures the fat and fibre content of the meal, respectively, in *grams*. Table 13 provides example nutrition data.

**Table 12:** UoM Nutrition Data Description.

Column	Type	Description	Units	Possible Values
meal_ts	Datetime	Datetime of observation	MM/DD/YYYY HH:MM	N/A
meal_type	String	Meal Type	N/A	Breakfast, Lunch, Dinner, Snack
meal_tag	String	Meal Tag	N/A	N/A
carbs_g	Int	Carbohydrates eaten	g	N/A
prot_g	Int	Proteins eaten	g	N/A
fat_g	Int	Fat eaten	g	N/A
fibre_g	Int	Fibre eaten	g	N/A

**Table 13:** Nutrition data example of UoM2301.

meal_ts	meal_type	meal_tag	carbs_g	prot_g	fat_g	fibre_g
22/10/2023 10:00	Breakfast	Coffee	0	0	1	0

### Activity data

The *UoMActivityID.csv* file captures activity-related information using twelve fields, as shown in Table 14. The *activity_ts* field records the precise datetime of each observation in the format *MM/DD/YYYY HH:MM*, enabling accurate tracking of activities. The *activity_type* field describes the type of activity, with possible values including *SEDENTARY*, *WALKING*, *RUNNING*, and *GENERIC*, where *GENERIC* refers to other forms of physical exertion not explicitly categorized—such as cycling, gym workouts, or swimming. The *active_Kcal* field quantifies the calories burned during active periods, measured in kilocalories *(Kcal)*. The *step_count* field records the number of steps taken, while the *distance_m* field measures the distance covered during the activity in meters. The *duration_s* field represents the total duration of the activity in *seconds*, complemented by the *active_time_s* field, which specifies the duration of active periods within the activity.

**Table 14:** UoM Activity Data Description.

Column	Type	Description	Units	Possible Values
activity_ts	Datetime	Datetime of observation	MM/DD/YYYY HH:MM	N/A
activity_type	String	Type of activity	N/A	SEDENTARY, WALK- ING, RUNNING, and GENERIC
active_Kcal	Int	Calories burned actively	kcal	N/A
step_count	Int	Steps taken	count	N/A
distance_m	Float	Distance covered	meters	N/A
duration_s	Int	Duration of activity	seconds	N/A
active_time_s	Int	Active time duration	seconds	N/A
start_time_s	Int	Activity start time	seconds	N/A
start_time_offset_s	Int	Start time offset	seconds	N/A
met	Float	Metabolic equivalent of task	METs	N/A
intensity	String	Intensity level	N/A	SEDENTARY, ACTIVE, HIGHLY_ACTIVE
motion_intensity_mean	Float	Mean motion intensity	N/A	N/A
motion_intensity_max	Float	Maximum motion intensity	N/A	N/A

**Fig. 3:**
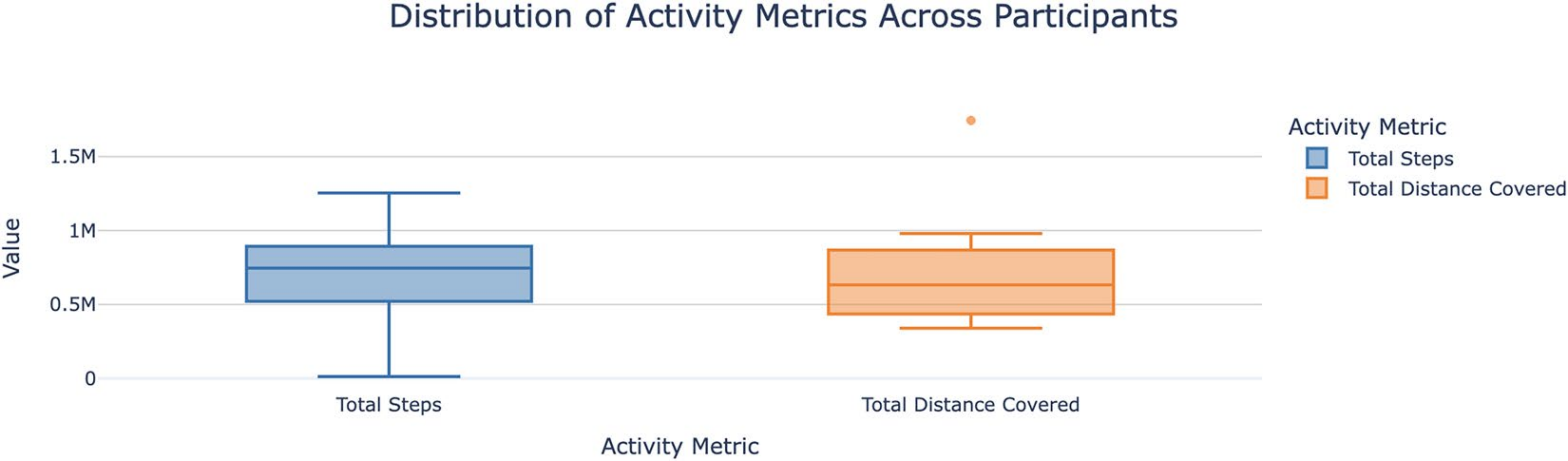
Distribution of activity metrics across participants. The figure presents the variability in physical activity levels among participants, measured in terms of step count and distance covered.

The *start_time_s* field denotes the start time of the activity in seconds since a reference point, and the *start_time_offset_s* field provides the offset from the reference start time. The *met* field indicates energy expenditure in METs, representing the intensity of physical activity relative to resting levels. The *intensity* field categorises the activity’s intensity level as *SEDENTARY*, *ACTIVE*, or *HIGHLY_ACTIVE*. Additionally, the *motion_intensity_mean* and *motion_intensity_max* fields measure the average and maximum motion intensity during the activity, respectively. Figure 3 illustrates the distribution of the total number of steps and distance covered by the participants. These variations highlight differences in individual lifestyles, fitness levels, and their potential effects on glucose metabolism. Understanding this distribution is essential for analysing how activity levels influence glycaemic control and insulin sensitivity in the management of T1D. Table 15 provides example activity data.

**Table 15:** Activity data example of UoM2301.

activity_ts	activity_type	active_Kcal	step_count	distance_m	duration_s	active_time_s
01/10/2023 05:45	SEDENTARY	0	0	0	900	900

start_time_s	start_time_offset_s	met	intensity	motion_intensity_mean	motion_intensity_max
1696135500	3600	1	SEDENTARY	0	2

### Sleep data

The *UoMSleepID.csv* file provides physiological and activity-related data through seven fields, as shown in Table 16. The *Timestamp* field captures the precise datetime of observation in the format *MM/DD/YYYY HH:MM:SS*, enabling detailed temporal analysis. The *heart_rate* field records the heart rate in beats per minute (*bPm*), offering insights into cardiovascular activity. The *curren_activity_type_intensity* field quantifies the intensity of the current activity as a count, while the *stress_level_value* field indicates the individual’s stress level on a scale. The *steps* field tracks the number of steps taken, serving as an indicator of physical activity during active periods.

**Table 16:** UoM Sleep Data Description.

Column	Type	Description	Units	Possible Values
Timestamp	Datetime	Datetime of observa- tion	MM/DD/YYYY HH:MM:SS	N/A
heart_rate	Int	Heart rate	beats per minute (bPm)	N/A
current_activity_type_intensity	Int	Current activity inten- sity	count	N/A
stress_level_value	Int	Stress level	scale	N/A
steps	Int	Steps taken	count	N/A
sleep_level	Int	Sleep/awake state	0/1	0/1
resting_heart_rate	Int	Resting heart rate	bPm	N/A

The *sleep_level* field represents sleep or awake status, with possible values of *0* for sleep and *1* for awake, facilitating the analysis of rest patterns. Lastly, the *resting_heart_rate* field measures the heart rate during rest in beats per minute (*bPm*), offering a baseline for understanding variations in heart activity. Table 17 provides example sleep data.

**Table 17:** Sleep data example of UoM2301.

sleep_ts	step_count	heart_rate
06/10/2023 06:55	19	76

current_activity_type_intensity	stress_level_value	sleep_level	resting_heart_rate
168	11	0	0

The *UoMsleeptime.csv* file provides more detailed and comprehensive sleep-related physiological data through fifteen fields, as shown in Table 18. The *calendar_date* field records the date of the sleep session in the format MM/DD/YYYY, enabling temporal analysis of sleep patterns. The *start_date_ts* field captures the precise start timestamp of sleep in MM/DD/YYYY HH:MM format, allowing for detailed time-based evaluations. The *duration_in_sec* field quantifies the total sleep duration in seconds, offering insight into overall sleep length.

**Table 18:** Sleep Time Data Columns and Descriptions.

Column	Type	Description	Units	Possible Values
calendar_date	Date	Calendar date of the sleep record	MM/DD/YYYY	N/A
duration_in_sec	Int	Total sleep duration	Seconds	N/A
start_date_ts	Datetime	Start timestamp of sleep	MM/DD/YYYY HH:MM	N/A
start_time_offset_s	Int	Offset from the start of the timestamp	Seconds	N/A
unmeasurable_sleep_s	Int	Time spent in unmeasur- able sleep states	Seconds	N/A
deep_sleep_s	Int	Duration spent in deep sleep	Seconds	N/A
light_sleep_s	Int	Duration spent in light sleep	Seconds	N/A
rem_sleep_s	Int	Duration spent in REM sleep	Seconds	N/A
awake_s	Int	Duration spent awake	Seconds	N/A
sleep_levels_map_deep	Object	Deep sleep segments	Object	Time intervals of deep sleep
sleep_levels_map_light	Object	Light sleep segments	Object	Time intervals of light sleep
sleep_levels_map_awake	Object	Awake segments	Object	Time intervals of wakefulness
sleep_levels_map_rem	Object	REM sleep segments	Object	Time intervals of REM sleep
sleep_levels_map_unmeasurable	Object	Unmeasurable segments	Object	Time intervals of unmeasurable sleep
validation	String	Sleep validation status	N/A	ENHANCED_FINAL, EN- HANCED_TENTATIVE, etc.

In contrast, the data presented in *UoMSleepID.csv* focuses on higher-frequency, real-time observations such as heart rate, step count, and binary sleep/awake states. This provides a broader yet less granular view of nightly sleep architecture compared to the staged breakdown offered by *UoMsleeptime.csv*.

The dataset *UoMsleeptime.csv* further categorizes sleep into distinct stages. The *deep_sleep_s, light_sleep_s, and rem_sleep_s* fields respectively capture the duration spent in deep sleep, light sleep, and REM sleep, all measured in seconds. Additionally, the *awake_s* field records the duration spent awake during the sleep session, facilitating the identification of wake periods. The *unmeasurable_sleep_s* field accounts for time intervals where sleep data could not be measured. To provide a structured representation of sleep cycles, the *sleep_levels_map_deep, sleep_levels_map_light, sleep_levels_map_rem, sleep_levels_map_awake, and sleep_levels_map_unmeasurable* fields contain time-segment mappings in object format, rep- resenting different sleep states at various timestamps. These mappings help in analysing sleep structure and transitions between sleep stages. Lastly, the validation field indicates the *validation* status of the sleep data, with possible values such as ENHANCED_FINAL and ENHANCED_TENTATIVE, signifying the reliability and accuracy of the recorded sleep session. Table 19 provides example sleep time data.

**Table 19:** Sleep time data example of UoM2301 containing general sleep data, sleep durations, and sleep level mappings.

calendar_date	duration_in_sec	start_date_ts	start_time_offset_s	unmeasurable_sleep_s
02/10/2023	33840	01/10/2023 20:51	3600	0

deep_sleep_s	light_sleep_s	rem_sleep_s	awake_s
4620	21480	7740	2340

sleep_levels_map.deep	sleep_levels_map.light	sleep_levels_map.awake
[{“startTimeInSeconds”:	[{“startTimeInSeconds”:	[{“startTimeInSeconds”:
1696193460, “endTimeIn-	1696193820, “endTimeIn-	1696193700, “endTimeIn-
Seconds”:1696193700},	Seconds”:1696194420},	Seconds”:1696193820},
…]	…]	…]

sleep_levels_map.rem	sleep_levels_map.unmeasurable	validation
[{“startTimeInSeconds”: 1696195980, “endTimeInSec- onds”:1696196160},…]		ENHANCED_FINAL

## Technical Validation

All data streams, including CGM (LibreView, Dexcom Clarity, CareLink), insulin delivery records, Garmin activity and sleep data, and nutritional logs, were timestamped by their respective devices. For 15 out of 17 participants residing in the United Kingdom, no time-zone conversion was necessary, as all devices were already synchronised to UK local time (GMT or BST, depending on the date). Timestamps were parsed and handled using Python’s pytz and datetime modules to ensure consistency across data modalities. Two participants (UoM2303 in Spain and UoM2320 in the Netherlands) remained abroad during their entire data collection period. Their devices were verified to be accurately synchronised with their respective local time zones, and as such, no adjustments were applied. Cross-modal temporal alignment was validated by checking for logical consistency across meal intake, insulin administration, glucose fluctuations, and physical activity. Garmin activity timestamps, initially in Unix epoch format, were converted to localised timestamps using participant-specific offsets. No clock drift or desynchronisation was identified.

### Blood glucose data

All authors collaboratively processed the raw data to produce a cleaned dataset, ensuring data integrity and consistency across participants. The cleaning process involved multiple steps. First, the raw files were parsed based on consistent participant identifiers and timestamps. Next, the authors removed duplicate entries, corrected formatting inconsistencies (e.g. improperly formatted numbers and timestamps), and handled missing or anomalous values using imputation or removal, depending on context and severity. For instance, physiologically implausible glucose readings (e.g., negative values or values outside biologically reasonable ranges) were cross-checked with adjacent measurements.

Each dataset underwent a thorough completeness check to ensure that all expected fields were present for each observation window. Furthermore, the authors performed an inter-rater reliability assessment on the blood glucose data by having multiple team members visually inspect the time series for outliers or inconsistencies. To further validate the process, glucose values were randomly sampled and compared between the raw and cleaned datasets, verifying that no data points were unintentionally omitted or altered during cleaning.

To further ensure the accuracy of the time-in-range (TIR) calculations, the computed values for each participant were systematically cross-validated against the raw glucose data. For instance, participant UoM2301 had a calculated TIR of 79%, which matched the corresponding value derived directly from the raw data for the same period. This validation confirmed the consistency and reliability of the TIR results across the dataset. Minor discrepancies were noted in a few cases; for example, participant UoM2313 had a reported TIR of 49% based on LibreView raw data from 18/01/2024 to 31/01/2024, while the calculated TIR for that same period was 50.77%. Such differences were minimal and fell within an acceptable margin, further reinforcing the integrity of the cleaned dataset.

### Activity data

To ensure the accuracy and reliability of the collected activity data, several validation steps were implemented. First, raw data from the Garmin Forerunner 45 was cross-checked against the data captured through the custom API to confirm synchronization and integrity. Additionally, a new column called *activity_ts* was created to convert the original *start_time_offset_s* (stored in Unix timestamp format) into a human-readable timestamp. To verify the integrity of this transformation, random timestamps were sampled for each participant and cross-checked against the original raw data to ensure the conversion was accurate. Sample data points from different time periods were manually reviewed and compared to the recorded activity logs to ensure that the activity data reflected the correct times and activities. Data completeness was also verified by checking that no critical periods of activity were missing from the collected dataset. These validation steps ensured both the accuracy and inter-rater reliability of the activity data.

**Fig. 4:**
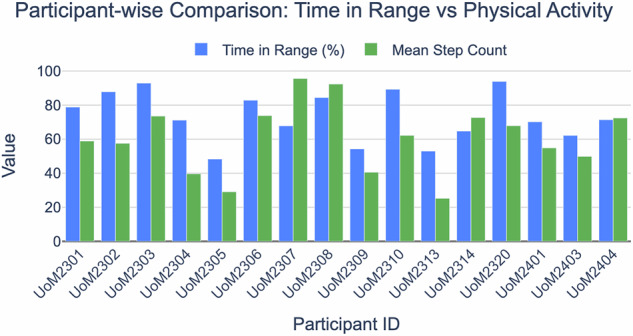
Participant-level variation in Time in range and daily step count. Higher physical activity did not consistently align with better glycaemic control, suggesting influence from additional factors such as insulin and nutrition.

The Fig. 4 illustrates the distribution of glycaemic control (TIR) and physical activity (daily step count) across participants. The inter-individual variability highlights the complex interplay between physical activity and glucose regulation, underscoring the need for personalised management strategies in T1D. Notably, higher physical activity did not uniformly translate to improved TIR, suggesting that additional factors such as insulin timing, nutrition, and individual insulin sensitivity may modulate these effects.

**Fig. 5:**
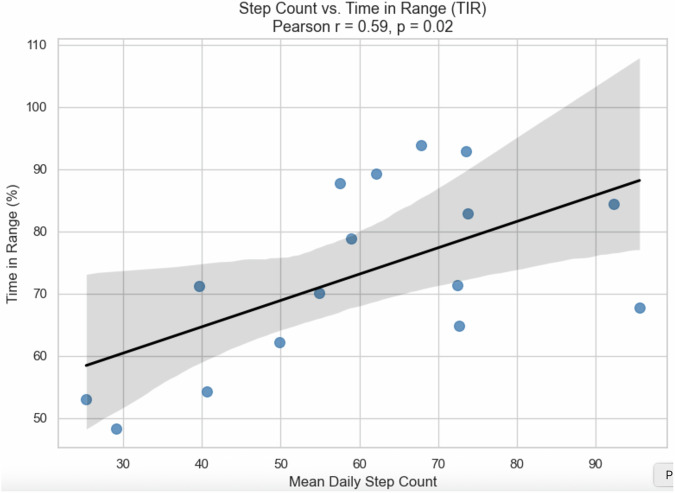
Positive correlation between mean daily step count and Time in Range (TIR). A moderate linear relationship was observed (Pearson r = 0.59, p = 0.02), suggesting increased physical activity may be associated with improved glycaemic control.

To quantify this relationship, a Pearson correlation analysis was conducted between mean daily step count and TIR across participants. The analysis revealed a moderate positive association (r = 0.59, p = 0.02), suggesting that higher levels of physical activity tended to align with higher TIR as show in Fig. 5.

### Sleep data

Sleep data were collected using the Garmin Forerunner 45, which employs motion detection and heart rate variability to estimate various sleep stages, including light, deep, and REM sleep. It should be noted that these devices, designed for general lifestyle monitoring and not as medical tools^31^, can have variable accuracy influenced by factors like device fit, the participant’s movements during sleep, and environmental conditions. Comparative studies show that under optimal conditions, Garmin’s sleep tracking is consistent with more specialized devices^32,33^. To verify the accuracy and reliability of our data, we compared sleep time and duration from participant-shared raw data with that retrieved from the API, finding no discrepancies, thus confirming the robustness of our data.

**Fig. 6:**
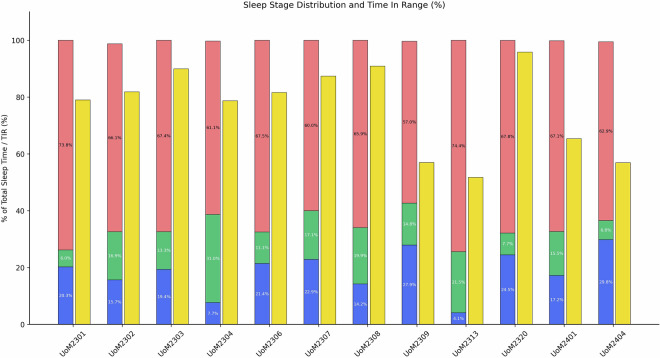
Bar chart shows each participant’s sleep stage distribution (Deep (blue), REM (green), and Light (red)) as stacked bars, alongside yellow bars representing time spent in target glucose range TIR (%).

**Fig. 7:**
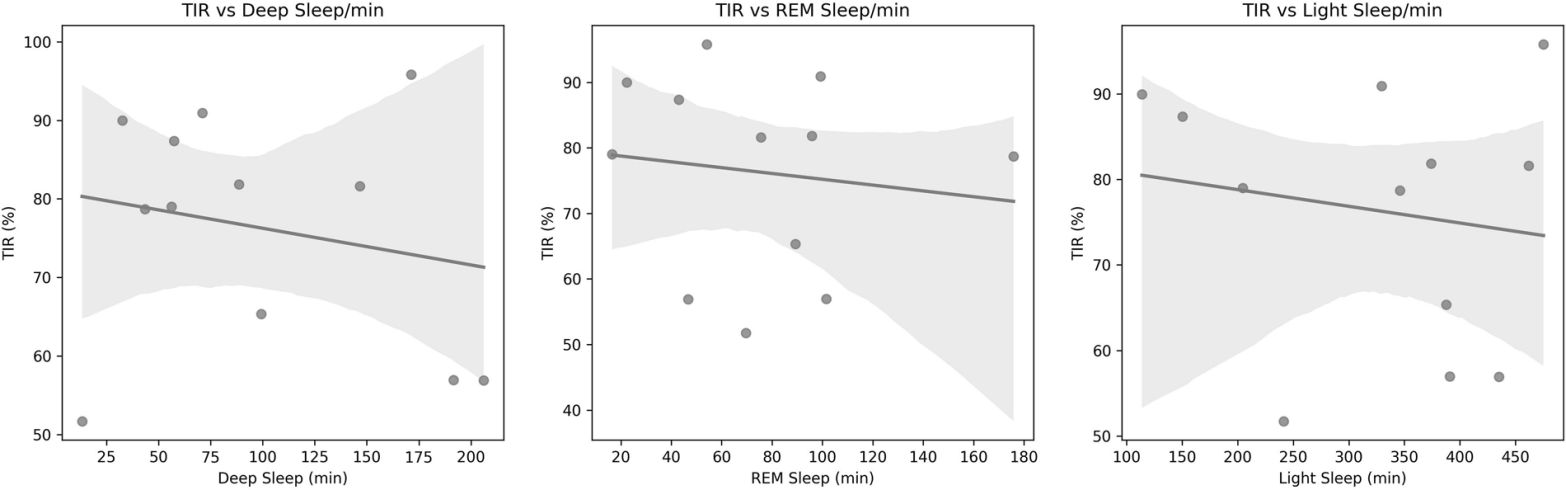
The associations between Time in Range (TIR%) and durations of Deep Sleep, REM Sleep, and Light Sleep (in minutes) across participants. Each subplot presents a scatterplot with a fitted regression line and confidence interval, showing that TIR% tends to decrease slightly with increased duration in individual sleep stages.

Table 20 presents key sleep metrics across participants, showing variability in total sleep time, mean sleep stages (REM, Deep, Light), and related physiological parameters such as mean glucose levels, sleep efficiency, and recorded stress level.The distribution of sleep stages as a percentage of total sleep time is presented in Fig. 6, alongside each participant’s TIR percentage, with sleep stages displayed as stacked bars and TIR represented by adjacent individual bars. Across the 12 participants, Light sleep included the largest segment of total sleep time in most individuals, while deep and REM sleep showed inter-individual differences. A negative correlation was observed between Time in Range (TIR %) and the different sleep stages as shown in Fig. 7.

**Table 20:** Sleep metrics summary per participant, including total sleep time (TST), mean sleep stages, mean glucose levels, and reported stress.

Subject	# Nights	TST (min)	REM (min)	Deep (min)	Light (min)	Awake (min)	Sleep Efficiency	Mean Glucose	Stress Level
UoM2301	31	418.81	25.26	90.23	303.32	6.10	97.41	8.18	21.63
UoM2302	68	413.99	5.93	78.12	329.94	84.51	97.67	7.86	12.82
UoM2303	22	405.91	6.23	89.18	310.50	61.09	97.67	7.48	10.59
UoM2304	70	344.24	37.20	296.57	10.47	150.73	95.99	7.98	12.68
UoM2306	85	432.53	3.02	103.52	325.99	53.40	98.79	7.67	11.21
UoM2307	30	432.03	16.53	114.80	300.70	85.93	93.71	7.50	12.61
UoM2308	66	166.44	33.50	23.23	109.71	7.15	92.97	6.98	12.05
UoM2309	77	467.71	13.75	149.31	304.65	79.14	95.13	9.69	12.65
UoM2313	30	648.83	26.63	482.87	139.33	18.10	94.46	9.64	19.43
UoM2320	84	465.98	4.21	122.38	339.38	38.62	98.39	7.26	8.50
UoM2401	71	486.79	75.38	83.97	327.44	21.42	92.13	7.57	15.80
UoM2404	79	492.89	5.96	156.52	330.41	35.47	97.78	9.11	25.19

### Insulin data

Descriptive statistics—such as mean and standard deviation—were calculated to summarize key variables in the dataset. These statistical summaries were then compared to the corresponding self-reported values provided by participants (Table 21) to assess the consistency and validity of the collected data. The analysis revealed that, in all instances where data were available, the extracted basal and bolus insulin values aligned with the self-reported values. However, comparisons could not be made for participants UoM2303, UoM2308, UoM2309, UoM2320, and UoM2404 due to missing collected or unreported data. These cases are indicated by’N/A’ in the corresponding rows and columns of the table. This absence of data may have been due to participants encountering issues with device synchronization or failing to provide the required data during the study period. This level of incompleteness (3^˜^0%) is consistent with limitations reported in other publicly available datasets such as HUPA-UCM^13^, Tidepool^14^, diaTribe^15^, and OhioT1DM^16^, where gaps in insulin logging are common due to irregular reporting or device syncing issues. Instead of excluding participants with insulin data that was incomplete or not non-comparable to self-reported data, these patients are retained to preserve the richness of the dataset, particularly because other variables such as sleep, nutrition, and physical activity remain complete and may be valuable for investigating additional Blood Glucose patterns from other standpoints. A particularly notable case is UoM2304, who transitioned to a closed-loop system during the data collection, resulting in the second half of their data being produced by this device. Furthermore, cases where the standard deviation of insulin doses was 0 *U* correspond to participants who exclusively used long-acting insulin, denoted as “L”, indicating a consistent daily dosage. In contrast, participants using rapid-acting insulin (“R”) exhibited greater variability in dosage and thus did not show this pattern of zero standard deviation.

**Table 21:** Comparison of self-reported and extracted insulin data: includes reported basal/bolus insulin, collected means and standard deviations, insulin type, delay between report and collection, and carbs/insulin ratio.

Participant ID	Basal Insulin Type	Reported Daily Basal [U]	Reported Daily Bolus [U]	Collected Basal Mean [U]	Collected Basal SD [U]	Collected Bo- lus Mean [U]	Collected Bo- lus SD [U]	Days Between Report and Collection	Carbs/Insulin Ratio
UoM2301	R	24	N/A	21.60	4.78	11.59	2.79	192	9–12
UoM2302	L	10	16	8.06	1.36	10.30	3.95	179	8–10
UoM2303	N/A	12	12	N/A	N/A	N/A	N/A	165	N/A
UoM2304	R	34.1	25–30	30.09	3.37	25.30	6.73	124	5–7
UoM2305	L	20	23	23.00	0.00	16.56	6.69	97	10
UoM2306	L	8	20–25	9.30	2.95	21.56	3.86	96	N/A
UoM2307	R	5–7	7–16	6.77	1.04	12.53	3.25	148	12–15
UoM2308	R	N/A	N/A	9.58	1.24	15.95	2.64	33	N/A
UoM2309	R	N/A	N/A	19.17	1.05	10.73	4.55	203	N/A
UoM2310	R	22	24	21.38	0.69	19.99	3.42	283	2.5
UoM2313	L	64	60	65.80	2.20	42.14	16.03	115	5
UoM2314	L	12	25	12.14	3.09	24.81	6.03	91	10
UoM2320	N/A	Variable	Variable	N/A	N/A	13.66	1.69	101	6–10
UoM2401	L	5–30	30	30.00	0.00	40.77	18.00	111	N/A
UoM2403	L	15	19	12.00	4.70	12.34	6.55	120	15
UoM2404	N/A	15	20	N/A	N/A	16.93	7.08	111	10
UoM2405	L	32	20	26.30	8.28	16.18	5.99	131	1.5

To further assess the reliability of the computed values, an additional column was introduced to capture the number of days between questionnaire completion and the final data collection. This interval varied significantly between participants, ranging from 33 to 283 days. A longer time gap may lead to greater variations in the mean and standard deviation of the values reported by participants. Therefore, this factor should be considered when interpreting the data.

### Nutrition data

**Fig. 8:**
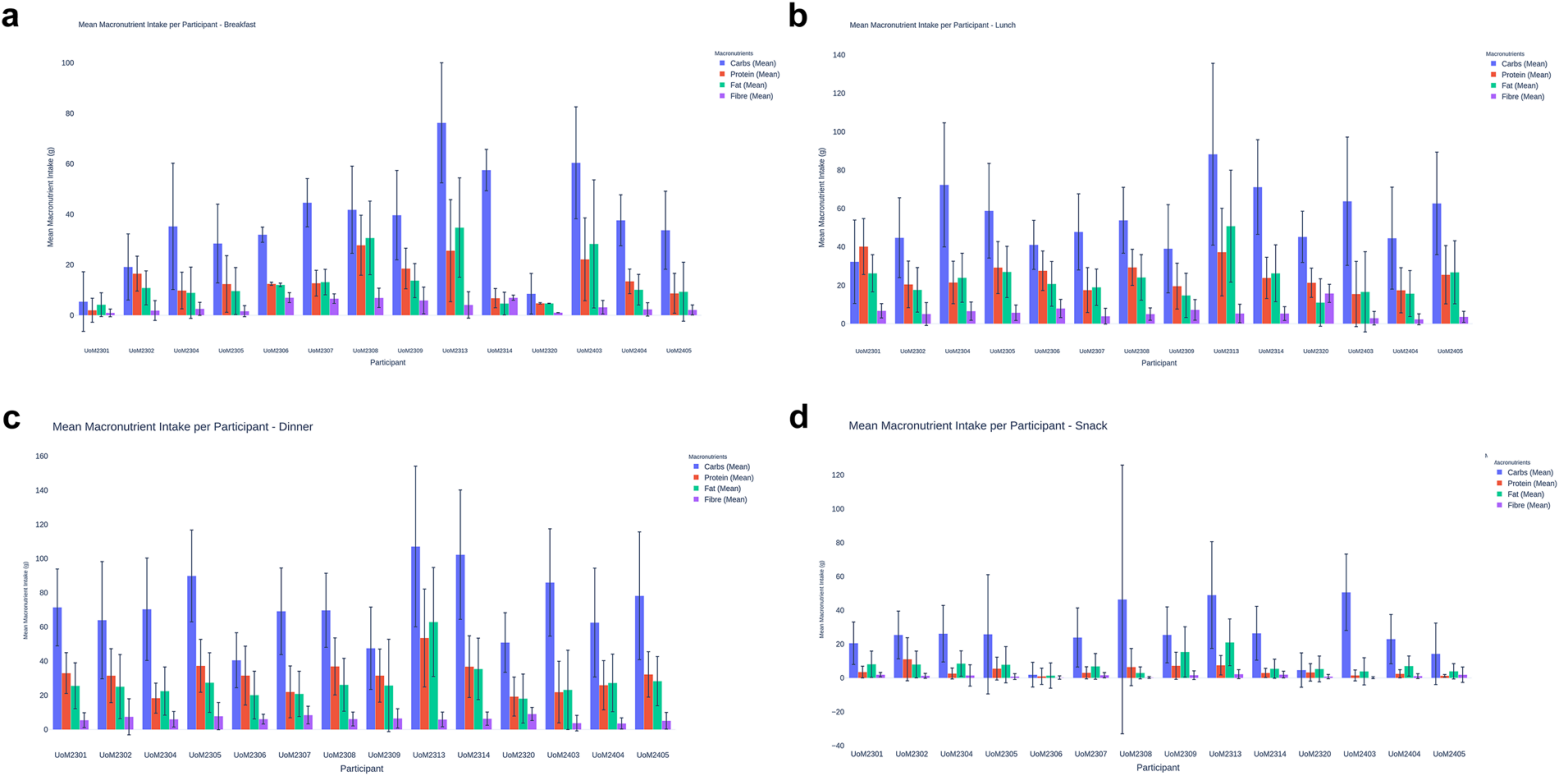
Macronutrient distribution per participant for each meal. (**a**) Breakfast. (**b**) Lunch. (**c**) Dinner. (**d**) Snack.

**Fig. 9:**
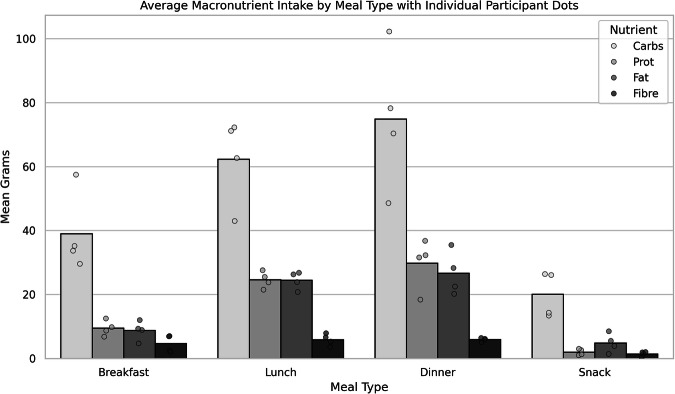
The bar plot visualizes the average intake of four key macronutrients, carbohydrates, protein, fat, and fiber, across different meal types: Breakfast, Lunch, Dinner, and Snack. Each bar represents the mean grams consumed for a specific nutrient within each meal category. Overlaid on the bars are jittered dots representing individual participant data points, allowing for visualization of variability and distribution around the mean values.

To ensure consistency in nutritional analysis across datasets, all food diary entries were standardized using Nutritics^22^. Given the inherent errors in self-reported food intake, including under-reporting, misreporting, and recall bias^34–36^, meal tags were used to cross-check nutritional composition when meals were reported multiple times. Self-reported nutritional data, while inherently limited, is often the only feasible option in real-world data collection. When used alongside objective measures like insulin dosing and individualized insulin-to-carb ratios, it provides a practical and contextually validated approach to estimating dietary intake and assessing data reliability. Nutritics was chosen for its comprehensive meal planning, recipe analysis, and nutrient tracking capabilities. Its advantages include a robust database, customizable reports, multi-language support, and integration with wearable devices, making it a reliable dietary assessment tool^37^. The Meal Tag system further strengthened this approach by correlating postprandial glucose responses (PPGR) with entire meals rather than isolated nutrient components. This method provides a more holistic understanding of glycaemic impact, considering factors beyond carbohydrate counting, such as gut microbiome composition, stress levels, and hormonal fluctuations. Two participants were excluded from the nutritional analysis: UoM2401, who failed to return their manual food diary, and UoM2312, whose dietary patterns significantly changed due to religious reasons, violating the inclusion criteria. The meal specific average nutritional intake across the dataset can be seen in Fig. 9. This is further analysed for each participant can be seen in Table 22 and in Fig. 8.

**Table 22:** Summary of macronutrient composition across meal types, including mean and standard deviation (Std) values for carbohydrates (g), protein (g), fat (g), and fiber (g), categorized by participant.

Participant ID	Meal Type	Carbs (g) Mean	Carbs (g) Std	Protein (g) Mean	Protein (g) Std	Fat (g) Mean	Fat (g) Std	Fibre (g) Mean	Fibre (g) Std
UoM2301	Breakfast	5.40	11.81	1.98	4.76	4.18	4.72	0.93	1.53
UoM2301	Dinner	71.46	22.49	33.00	11.94	25.58	13.41	5.51	4.33
UoM2301	Lunch	32.25	21.75	40.19	14.57	26.26	9.65	6.73	3.72
UoM2301	Snack	20.54	12.57	3.48	3.46	8.14	7.78	1.94	1.28
UoM2302	Breakfast	19.13	13.11	16.49	6.91	10.84	6.73	1.87	3.91
UoM2302	Dinner	63.98	34.28	31.50	15.76	25.12	18.74	7.45	10.54
UoM2302	Lunch	44.78	20.82	20.47	12.16	17.63	11.60	5.06	5.94
UoM2302	Snack	25.40	14.11	11.07	12.73	8.00	7.90	1.27	1.44
UoM2304	Breakfast	35.22	25.03	9.79	7.27	8.92	10.14	2.54	2.57
UoM2304	Dinner	70.39	29.96	18.37	8.81	22.50	14.06	6.02	4.56
UoM2304	Lunch	72.33	32.33	21.48	11.08	23.94	12.75	6.56	4.75
UoM2304	Snack	26.14	16.74	2.64	3.21	8.53	7.51	1.48	6.32
UoM2305	Breakfast	28.42	15.62	12.39	11.22	9.61	9.29	1.61	2.19
UoM2305	Dinner	89.86	26.86	37.25	15.46	27.47	17.42	7.83	7.96
UoM2305	Lunch	58.83	24.65	29.24	13.55	26.98	13.33	5.71	4.01
UoM2305	Snack	25.79	35.25	5.53	6.75	7.84	10.70	0.84	1.71
UoM2306	Breakfast	31.91	3.02	12.45	0.62	12.03	0.69	6.99	1.97
UoM2306	Dinner	40.55	16.08	31.56	17.22	20.16	13.95	6.12	2.87
UoM2306	Lunch	41.06	12.76	27.58	10.30	20.75	11.67	7.93	4.74
UoM2306	Snack	1.95	7.30	0.96	4.81	1.40	7.46	0.24	0.99
UoM2307	Breakfast	44.60	9.56	12.70	5.10	13.14	5.05	6.58	1.91
UoM2307	Dinner	69.18	25.34	22.02	15.17	20.84	13.23	8.49	5.17
UoM2307	Lunch	47.83	19.82	17.50	11.70	19.00	9.48	3.92	4.09
UoM2307	Snack	23.92	17.46	3.05	3.55	6.84	7.60	1.62	1.56
UoM2308	Breakfast	41.80	17.28	27.73	11.90	30.64	14.59	6.90	3.84
UoM2308	Dinner	69.76	21.68	36.91	16.68	26.15	15.46	6.15	4.06
UoM2308	Lunch	53.86	17.17	29.32	9.41	24.11	11.88	5.01	3.21
UoM2308	Snack	46.43	79.42	6.43	11.00	3.00	3.51	0.29	0.49
UoM2309	Breakfast	39.67	17.69	18.51	8.03	13.72	6.74	5.86	5.29
UoM2309	Dinner	47.53	24.08	31.55	15.53	25.78	27.00	6.53	5.63
UoM2309	Lunch	39.05	22.97	19.55	11.95	14.74	11.52	7.22	5.35
UoM2309	Snack	25.45	16.52	7.20	7.99	15.40	14.86	1.66	2.56
UoM2309	Supper	44.30	24.95	18.67	13.46	18.11	14.27	8.39	6.53
UoM2313	Breakfast	76.25	23.79	25.58	20.22	34.75	19.71	4.08	5.23
UoM2313	Dinner	107.07	46.99	53.56	28.63	62.92	31.89	5.90	4.33
UoM2313	Lunch	88.27	47.37	37.29	22.83	50.82	29.17	5.32	4.80
UoM2313	Snack	49.02	31.57	7.51	5.77	21.05	13.85	2.27	2.64

Participant ID	Meal Type	Carbs (g) Mean	Carbs (g) Std	Protein (g) Mean	Protein (g) Std	Fat (g) Mean	Fat (g) Std	Fibre (g) Mean	Fibre (g) Std
UoM2314	Breakfast	57.53	8.20	6.78	3.82	4.65	4.48	6.95	1.01
UoM2314	Dinner	102.30	37.84	36.78	18.03	35.46	17.99	6.36	3.88
UoM2314	Lunch	71.17	24.67	23.85	10.77	26.26	14.79	5.32	3.58
UoM2314	Snack	26.42	15.91	2.96	2.71	5.47	5.64	2.01	1.98
UoM2320	Breakfast	8.52	8.02	4.73	0.28	4.68	0.10	1.01	0.04
UoM2320	Dinner	50.89	17.42	19.29	11.40	18.10	14.36	9.11	3.82
UoM2320	Lunch	45.25	13.36	21.33	7.61	11.04	12.37	15.79	4.72
UoM2320	Snack	4.68	10.14	3.28	5.14	5.33	7.64	0.82	1.28
UoM2403	Breakfast	60.40	22.16	22.15	16.41	28.25	25.38	3.18	2.67
UoM2403	Dinner	86.04	31.41	21.90	18.02	23.21	23.21	3.76	4.57
UoM2403	Lunch	63.82	33.42	15.48	17.00	16.61	20.90	2.91	3.49
UoM2403	Snack	50.67	22.61	1.50	3.22	3.89	8.03	0.17	0.51
UoM2404	Breakfast	37.62	10.11	13.42	4.88	10.10	6.07	2.33	2.64
UoM2404	Dinner	62.59	31.86	25.94	14.38	27.28	16.84	3.58	3.25
UoM2404	Lunch	44.57	26.57	17.41	11.75	15.70	11.96	2.31	2.83
UoM2404	Snack	22.96	14.57	2.47	2.40	7.04	5.97	1.09	1.42
UoM2405	Breakfast	33.68	15.47	8.69	7.90	9.33	11.69	2.09	1.98
UoM2405	Dinner	78.25	37.39	32.27	13.27	28.33	14.40	5.15	4.86
UoM2405	Lunch	62.68	26.70	25.50	15.18	26.76	16.42	3.58	2.87
UoM2405	Snack	14.27	18.19	1.27	0.80	3.93	4.50	1.93	4.51

In instances where self-reported meal or insulin data was missing, PPGR initiation times can be imputed based on matched events with similar contextual features, specifically, by aligning with entries from the same day of the week and similar meal type. The imputation process can use the closest available data point that reflected the average pattern of comparable matched events, ensuring contextual relevance. For example, a missing Monday breakfast entry from week 2 could be imputed by referencing isolated Monday breakfast data from weeks 1 and 3.

Preprocessing scripts used to isolate and model PPGRs, including steps for addressing missing or inconsistent logging, can be found at the Zenodo repository^30^ ‘T1D-UOM – A Longitudinal Multimodal Dataset of Type 1 Diabetes’ at 10.5281/zenodo.15806142.

### Limitation

One potential limitation of this study is the relatively small number of participants (n = 17), which may limit the generalizability of findings, particularly for applications that rely on large and diverse training populations. However, the dataset provides dense, high-resolution multimodal data per individual—including glucose, insulin (basal and bolus), nutrition, activity, and sleep—collected continuously over a 12-week period. This richness supports the development of machine learning models that leverage temporal and contextual detail, such as recurrent neural networks (RNNs), transformer-based models, or personalized reinforcement learning approaches. These models can benefit significantly from the volume and granularity of data available per person, enabling them to learn complex intra-individual patterns relevant to T1D management. Future work will expand the cohort to include broader demographic diversity.

## Data Availability

The code used to access and process the Garmin API data is also publicly available on the Zenodo repository at 10.5281/zenodo.14961985.
